# A comparative study on hypoglycemic properties, lipid profile and bioactive components of hydro-alcoholic extracts of cooked and raw *Brassica napus*

**Published:** 2015-08-15

**Authors:** Fatemeh Akbari, Samaneh Khodadadi, Sedigheh Asgari, Hedaytolah Shirzad, Mahmoud Mirhoseini, Najmeh Shahinfard, Mahmoud Rafieian-Kopaei

**Affiliations:** ^1^Medical Plants Research Center, Shahrekord University of Medical Sciences, Sharekord, Iran; ^2^Nickan Research Institute, Isfahan, Iran; ^3^Isfahan Cardiovascular Research Center, Cardiovascular Research Institute, Isfahan University of Medical Sciences, Isfahan, Iran

**Keywords:** Diabetes mellitus, *Brassica napus*, Insulin

## Abstract

**Introduction:** Many plants with anti-oxidant properties proved to be effective on diabetes treatment. *Brassica napus* (turnip) is an anti-oxidant plant consumed raw or cooked. In this study, we examined and compared hypoglycemic and hypolipidemic properties of raw and cooked *Brassica napus* in diabetic rats.

**Objectives:** Due to measuring bioactive component of *Brassica napus* as a rich source of flavonoid we investigate the hypoglycemic properties in raw and cooked type.

**Material and Methods:** For this experimental study, 50 male Wistar rats weighing 200-250 g were designated into five groups of 10 consist of control, diabetic control, diabetic cooked turnip, diabetic raw turnip, and diabetic glibenclamide. The alloxan-induced diabetic rats received extracts orally for 4 weeks. Then, the serum biochemical factors were measured and compared statisticaly by analysis of variance (ANOVA) test.

**Results:** Serum glucose, triglyceride (TG), cholesterol, and low density lipoprotein cholesterol (LDL-C) were significantly decreased in cooked and raw turnip rats compared to control ones. Cooked and raw *Brassica napus* extracts both helped high density lipoprotein cholesterol (HDL-C) increase; cooked turnip competency was superior in view of cholesterol and LDL-C decrease as well as HDL-C increase (P < 0.05). The mean difference in glucose and TG decrease was not significant between diabetic cooked turnip and diabetic raw turnip rats.

**Conclusion:** Improving the blood glucose and lipid levels diabetic rats, in this study, may indicate that both raw and cooked *Brassica napus* extracts (especially the cooked one) may be beneficial in diabetic patients.

**Implication for health policy/practice/research/medical education:**

Brassica napus (turnip) is an anti-oxidant plant probably be effective in treatment of diabetes in raw or cooked form.

## Introduction

According to the World Health Organization (WHO), nowadays, 150 million people in the world are suffering from type II diabetes mellitus and this number will increase to double by 2025. The mortality rate of diabetic people is 1.5 to 2.5 times higher than the general population. In most of the cases, type II diabetes and insulin resistance are associated with hyperglycemia and obesity (1). Overweight and obesity are also considered as the main causes of diabetes and insulin resistance. It has been documented that disturbed lipid profile is usually observed in patients or animals suffering diabetes mellitus. Lipid peroxidation and consequent oxidative stress are increased in diabetes and hyperlipidemia, and have been proposed to play roles in the pathogenesis of these diseases. Furthermore, increase in adipose tissue, particularly visceral adipose, is strongly associated with development of diabetes and metabolic syndrome (1,2). Adipose tissue is an endocrine tissue which secrets some proteins such as adipokines mostly affecting lipid and glucose metabolism, as well as insulin resistance; whereas some of them as adiponectin have protective effect against insulin resistance. Visfatin is a new adipocytokine mainly secreted by visceral adipose tissue with similar effects of insulin, and a possible role in development of diabetes and inflammatory reactions. Clinical studies have demonstrated the direct association of higher circulating levels of visfatin with body fat percentage, type II diabetes and obesity (2).

Diabetes mellitus is one of the most important clinical risk factors for some disorders, e.g. nephropathy, neuropathy, retinopathy, and cardiovascular diseases. Although insulin and oral antidiabetic drugs are widely used for the treatment of diabetes, a nutritional or traditional approach could yield better outcomes (3).

These plants have always been paid attention as suitable substitutes for chemical drugs due to easy accessibility, less side effects, low toxicity, and cost-effectiveness, exhibiting reportedly helpful effects. *Brassica napus* (Turnip) from Brassicaceae family is helpful for many complications such as renal disorders, cystitis, gout, joint pain, abscess, hypertension and rheumatoid arthritis. Abundantly present in *Brassica napus*, flavonoids and cinnamic acid hydroxyl derivatives, have also direct and marked anti-oxidant activities. *Brassica napus* is also rich in anthocyanin, another compound with anti-oxidant features (4). Given the material available, the plant is expected to be effective on diabetes treatment regarding anti-oxidant activities and the presence of some compounds, e.g. flavonoids.

It has been demonstrated that cooking may change the therapeutic activity of medicinal plants. Since *Brassica napus* is consumed both raw and cooked, this study was aimed to compare the raw and cooked effects of this plant on serum glucose and lipids in rats. Also, the level of phenolic and flavonoid compounds as well as the anti-oxidant activities of these two forms were measured and compared.

## Objectives

The positive effect of many medicinal plants on glucose and lipid decrease or their complications has so far been recognized. In this study we determined to investigate on hypoglycemic properties, lipid profile and bioactive components of hydro-alcoholic extracts of cooked and raw *Brassica napus* as high antioxidant potential.

## Materials and Methods

### Extraction

Having been approved by a botanist, a herbarium sample of *Brassica napus* (No. 325, Herbarium Unit, Shahrekord University of Medical Sciences) grown in Isfahan, Iran was provided. The Turnip was carefully washed, dried in the shade, and its juice was obtained. Then the juice was concentrated to 1/3 of the original volume by a rotary evaporator at 45°C and the rotation speed of 70 rpm. For the cooked plant, it was fully cooked for 20 minutes and the juice was obtained. Afterwards, the cooked *Brassica napus* was boiled twice and the juice was again obtained and added to previous one. At the end it was concentrated per procedure described for the raw plant. To standardize, the phenolic and flavonoidic components as well as the anti-oxidant activity of both extracts were measured as follows.

### Measuring phenolic compounds

The level of phenolic compounds were measured based on Sharafati et al method. Briefly, 0.5 ml Folin-Ciocalteu was added to 0.1 ml of the diluted extract (0.01 g of the extract in 10 ml methanol 60%) (5). After 3-5 minutes 0.4 ml sodium carbonate 7.5% was introduced into the solution. Then, the absorption was read against distilled water blanching after 30-minute incubation at the room temperature. Concurrently, different dilutions of gallic acid were prepared and tested to develop standard curve. The level of samples’ absorption was compared against the standard curve and the total phenolic compounds were measured in mg per 1 g of the dried extract.

### Measuring total flavonoid compounds

Shortly speaking 0.5 ml of each extract (0.01 g of the extract in 10 ml methanol 60%) was added to 0.5 ml aluminum chloride 2%, and 3 ml potassium acetate 5% introduced into the solution. After 40 minutes the samples’ absorption was read against distilled water at 415 nm wavelength. Samples’ absorption was compared against the standard curve and each extract’s flavonoid measured in milligram per gram of the dried extract.

### Measuring total flavonol compounds

For this 0.5 ml of each extract (0.01 g of the extract in 10 ml methanol 60%) dissolved with 0.5 ml aluminum chloride 2% was added to 3 ml sodium acetate 5%. After 2.5 hours, absorption was read against distilled water at 440 nm wavelength. Samples’ absorption was compared against the standard curve and the total flavonols were measured in milligram per gram of the extract. Meanwhile different rutin concentrations were prepared and tested to develop the standard curve.

### Measuring the anti-oxidant activity

To do this, Shirzad et al method (6) was adopted with a minor change. In this method, 0.5 ml beta-carotene was dissolved with 1 ml chloroform. To this emulsion, 25 µl linoleic acid and 200 mg tween 40 was added. Then, 100 ml oxygen-saturated water (under 100 ml/min pressure for 30 minutes with vigorous shakings) was also added. The targeted extracts were prepared at 2 mg/l concentration in ethanol. Two and a half ml of the prepared solution was, separately, added to 350 µl of the prepared extract and butylated hydroxytoluene (BHT) (as control). The anti-oxidant activity was measured in terms of beta-carotene colour removal at 490 nm wavelength within 48 hours using the following formula:

AA = 100 [1-(A_o_- A_t_)/(A^o^_o_ – A^o^_t_)],

where A_o_ and A^o^_o_ represent optical absorption at A_t_ and A^o^_t_ respectively within 48 hours for cases and control; the mean was determined as percentage of anti-oxidant activity. The results obtained for phenolic, flavonol, and flavonoidic compounds, and anti-oxidant activity were expressed as mean ± SD.

### Effects of the extracts on glucose and lipid profile

Fifty male rats weighing 200-250 g were supplied from Pasteur Institute of Iran and randomly designated into five 10-member groups; 1. control, 2. diabetic control (DC), 3. diabetic cooked turnip, 4. diabetic raw turnip, and 5. diabetic glibenclamide (DG), as positive control group. The control rats were fed a normal diet for four weeks. The diabetic cooked and raw turnip rats, for the same period, received respectively cooked and raw Brassica napus extract orally in 16 ml per kilogram of body weight (kg/BW) (3 g/kg dried extract).

### Making the animals diabetic

The rats were made diabetic by alloxan monohydrate dissolved in normal saline which was intraperitoneally administered in 120 mg/kg BW for three consecutive days.

### Biochemical measurements

A glucometer (Match Glucose Monitoring System, Taiwan) was used to confirm diabetes. For this, a blood drop which was taken from rat’s tail by a lancet 3 days after alloxan monohydrate administration was placed on the paper strip and the glucose was read. To report blood glucose an enzymatic method (BioSystems, Pars Azmoon kit, Iran) was adopted.

Measurement of triglyceride (TG) and cholesterol was performed also by the enzymatic method (BioSystems, Pars Azmoon kit, Iran). Low density lipoprotein cholesterol (LDL-C) was calculated by: LDL-C = Total cholesterol - ( HDL + TG/5 )

### Data analysis

Comparing mean levels of the investigated factors was done through analysis of variance (ANOVA) using SPSS 11.5.* P*<0.05 was considered as statistically significant. For drawing graphs Excel software was employed.

## Results

### Comparing effects on serum glucose

At the beginning of the study, no significant difference was observed among the groups. Administering alloxan monohydrate for three consecutive days induced diabetes and the rats’ blood glucose initially 96 ± 70 mg/kg BW increased to 489 ± 310 mg/kg BW.

**Figure 1:**
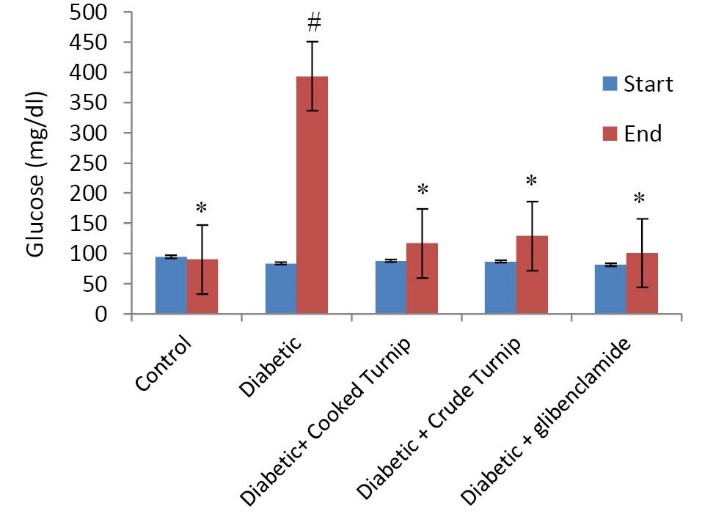
Comparison of blood glucose. The results were presented as Mean ± SE (n = 10). *P < 0.05, compared to the controls during the same period. #^#^*P* < 0.05, at the end compared to at the beginning as well as the controls.

Both raw and cooked extracts helped serum glucose significantly decrease compared to diabetic control rats (*P*<0.05). In this regard, no significant difference was observed between diabetic raw and cooked turnip, diabetic raw turnip and diabetic glibenclamide, as well as diabetic cooked turnip and diabetic glibenclamide rats (Figure 1).

### Comparing effects on serum triglyceride level

**Figure 2:**
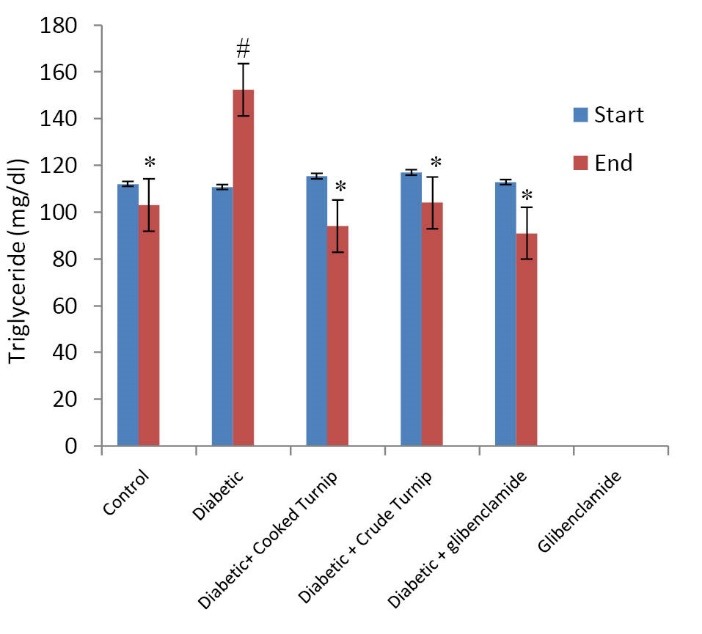
Comparison of blood TG. The results were presented as Mean ± SE (n = 10). *P < 0.05, compared to the controls during the same period. ^#^*P* < 0.05, at the end compared to at the beginning as well as the controls.

No significant difference in serum TG was observed among the groups. Diabetes made serum TG which was initially 90 mg/dl increase to 390 mg/dl. Consuming the raw and cooked extracts both decreased serum TG when compared to diabetic control rats (P<0.05). There was no significant difference between the raw and cooked extracts (Figure 2).

### Comparing effects on serum cholesterol

**Figure 3:**
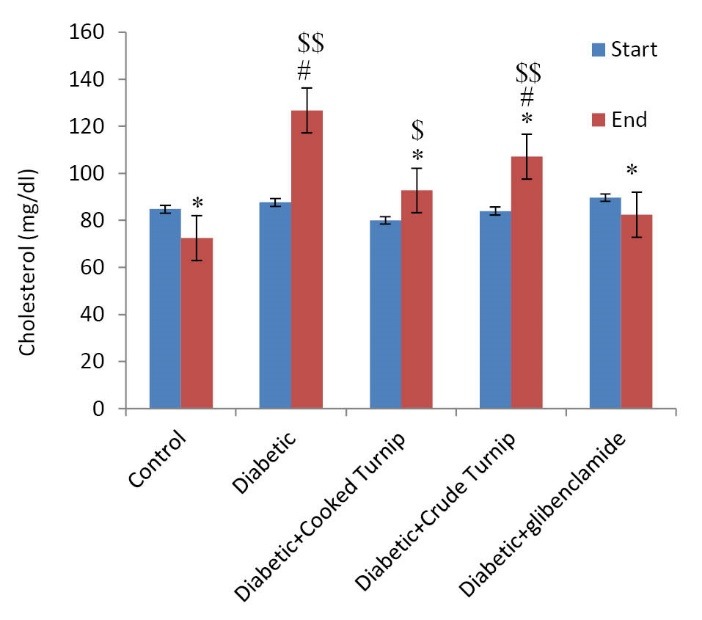
Comparison of serum cholesterol. The results were presented as Mean ± SE (n = 10). *P < 0.05, compared to the controls during the same period. ^#^*P* < 0.05, at the end compared to at the beginning as well as the controls. ^$^*P* < 0.05, diabetic cooked turnip rats compared to diabetic raw turnip and control rats. ^$$^*P* < 0.05, compared to diabetic glibenclamide during the same period.

Consuming the raw and cooked extracts decreased serum cholesterol significantly compared to diabetic control rats (*P*<0.05). As illustrated, the cooked extract in contrast to the raw extract helped cholesterol decrease more significantly (*P*<0.05). No significant difference was observed between diabetic raw turnip and diabetic glibenclamide as well as diabetic cooked turnip and diabetic glibenclamide rats (*P*<0.05) (Figure 3).

### Comparing effects on serum HDL-C

**Figure 4:**
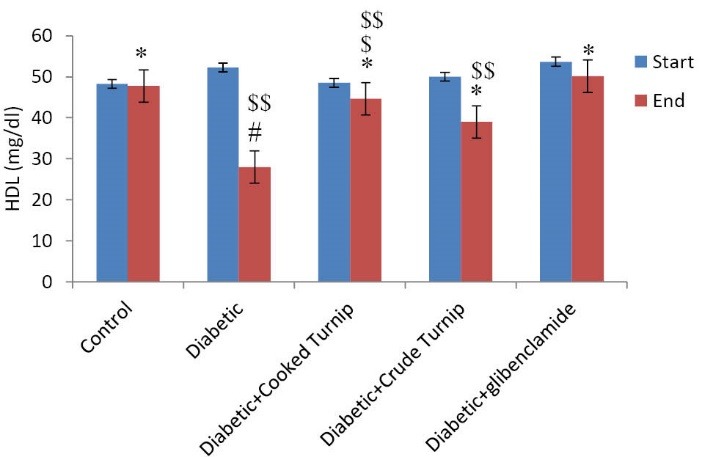
Comparison of serum HDL-C. The results were presented as Mean ± SE (n = 10). *P < 0.05, compared to the controls during the same period. #^#^*P* < 0.05, at the end compared to at the beginning as well as the controls. ^$^*P* < 0.05, diabetic cooked turnip rats compared to diabetic raw turnip and control rats. ^$$^*P* < 0.05, compared to diabetic glibenclamide during the same period.

Figure 4 illustrates that there is a significant difference in mean HDL-C between diabetic cooked turnip and DC, and diabetic raw turnip and DC rats, i.e., it is higher in diabetic cooked turnip and diabetic raw turnip compared to DC rats. Also there is a significant difference between diabetic raw turnip and diabetic cooked turnip rats, indicating the cooked extract increased HDL-C more significantly compared to the raw extract while glibenclamide helped HDL-C increase more significantly compared to both raw and cooked extracts.

### Comparing effects of serum LDL-C

**Figure 5:**
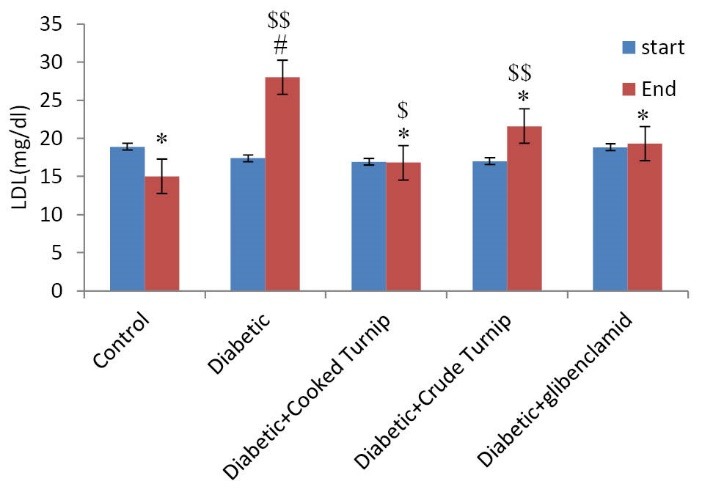
Comparison of serum LDL-C. The results were presented as Mean ± SE (n = 10). *P < 0.05, compared to the controls during the same period. #^#^*P* < 0.05, at the end compared to at the beginning as well as the controls. ^$^*P* < 0.05, diabetic cooked turnip rats compared to diabetic raw turnip and control rats. ^$$^*P* < 0.05, compared to diabetic glibenclamide during the same period.

Both raw and cooked extracts helped serum LDL-C decrease, more significantly in diabetic cooked turnip rats compared to diabetic raw turnip and DG rats (*P*<0.05) (Figure 5).

## Discussion

The present study was conducted to compare the raw and cooked *Brassica napus* extract effects on glucose and lipid profile in diabetic rats. The extract phenolic and flavonoid compounds, and anti-oxidant activities were also evaluated. Alloxan monohydrate contributing to the production of active oxygen species in pancreas and destruction of Langerhans cells was employed to experimentally induce diabetes in animals, which led to increased glucose (7). One of the reasons for alloxan-induced increase in glucose is pancreas destruction and hence loss of insulin secretion which in turn activates phosphorylase; this enzyme causes glycogen breakdown into glucose phosphate. On the other hand, glucose phosphatase is, due to insulin loss, activated which helps phosphate separate from glucose, allowing it to again enter into bloodstream from liver (1,3).

Regarding our results the raw and cooked *Brassica napus* extracts could help glucose decrease significantly in diabetic rats compared to DC rats (4,8). Cartea et al in 2011 found that the plants belonging to Brassicaceae family, like *Brassica napus*, are rich in anthocyanins including pelargonidin, cyanidin, delphinidin, peonidin, petunidin, and malvidin. Anthocyanins have some role in decreasing and/or inhibiting alpha-glucosidase, which prevents carbohydrate hydrolysis, resulting in decrease in glucose concentration (9). Furthermore, anthocyanin-containing compounds induce insulin secretion via stimulating pancreas β-cells, probably effective on prevention and treatment of diabetes type 2 (3). *Brassica napus* also has sulfur-containing amino acids. Reported to directly have glucose lowering function, enhance insulin effect on body, and increase liver glycogen synthesis in diabetic mice, rats and rabbits. Our study was indicative of high levels of flavonoids in *Brassica napus*, potentially making this plant effective on diabetes treatment.

Following alloxan monohydrate administration TG and cholesterol, in addition to glucose, increased; diabetes reportedly has been associated with marked and unpleasant variations in plasma lipids. In this regard, alloxan- and streptozotocin-caused elevation of TG and cholesterol has already been confirmed. In addition serum glucose elevation, in rats made diabetic by alloxan monohydrate, could indirectly contribute to increase in serum cholesterol, TG, and LDL-C, and decrease in HDL-C (2).

Brassica napus extract, raw and cooked, caused plasma cholesterol level decrease significantly, higher in case of consuming the cooked plant extract. *Brassica napus* rich in anthocyanins induces the reverse cholesterol flow from macrophages, leading to cholesterol absorption from circulation and its storage in the liver (4). Based on our findings the raw and cooked *Brassica napus* extracts both are full of flavonoidic compounds. Madani et al indicated that those plant extracts that are rich in flavonoids play some role, owing to anti-oxidant activities, in cholesterol decline. The significant difference in two groups could be explained by the finding according to which flavonoids and phenolic compounds were, in our experiments, higher in the cooked compared to the raw turnip extract (10).

Here the raw and cooked *Brassica napus* extracts helped serum TG decrease significantly. Anthocyanins and other anti-oxidant compounds are able to pronouncedly decrease lipids accumulation in the liver (2).

Flavonoids and phenolic compounds decreased hyperlipidemia in diabetic rats, improved lipid peroxidation, declined LDL-C, and increased HDL-C. Higher decrease in LDL-C and greater increase in HDL-C could be attributable to higher levels of flavonoids and phenolic compounds in the cooked plant extract (2,3,9).

## Conclusion

Consuming *Brassica napus*, raw and cooked, could decrease glucose and lipid in a positive manner. However, the cooked plant compared to the raw plant is possibly more helpful for patients with diabetes and hyperlipidemia considering the higher significant effect of the cooked plant on serum cholesterol, HDL-C, and LDL-C.

## Acknowledgements

We are thankful for the Research and Technology Deputy of Shahrekord University of Medical Sciences for supporting MSc thesis of Fatemeh Akbari (Grant no. 928).

## Authors’ contribution

All authors contributed to design of the research. FA, SA, NSH and RA wrote the manuscript. MRK and SKH edited the paper. All authors read and approved the paper.

## Conflicts of interest

The authors declared no competing interests.

## Ethical considerations

Ethical issues (including plagiarism, data fabrication, and duplicate publication) have been completely observed by the authors.

## Funding/Support

This study was obtained from MSc thesis of Fatemeh Akbari funded by Research and Technology Deputy of Shahrekord University of Medical Sciences (Grant no. 928).
